# Controlled Structuring of Hyaluronan Films by Phase Separation and Inversion

**DOI:** 10.1021/acs.langmuir.3c01547

**Published:** 2023-09-01

**Authors:** Petr Smolka, Markéta Kadlečková, Karolína Kocourková, Martina Bartoňová, Filip Mikulka, Eliška Knechtová, Aleš Mráček, Lenka Musilová, Martin Humenik, Antonín Minařík

**Affiliations:** † Department of Physics and Materials Engineering, Tomas Bata University in Zlín, Vavrečkova 5669, Zlín 760 01, Czech Republic; ‡ Centre of Polymer Systems, Tomas Bata University in Zlín, Třída Tomáše Bati 5678, Zlín 760 01, Czech Republic; § Department of Biomaterials, Faculty of Engineering Science, Universität Bayreuth, Prof.-Rüdiger-Bormann.Str. 1, Bayreuth 95447, Germany

## Abstract

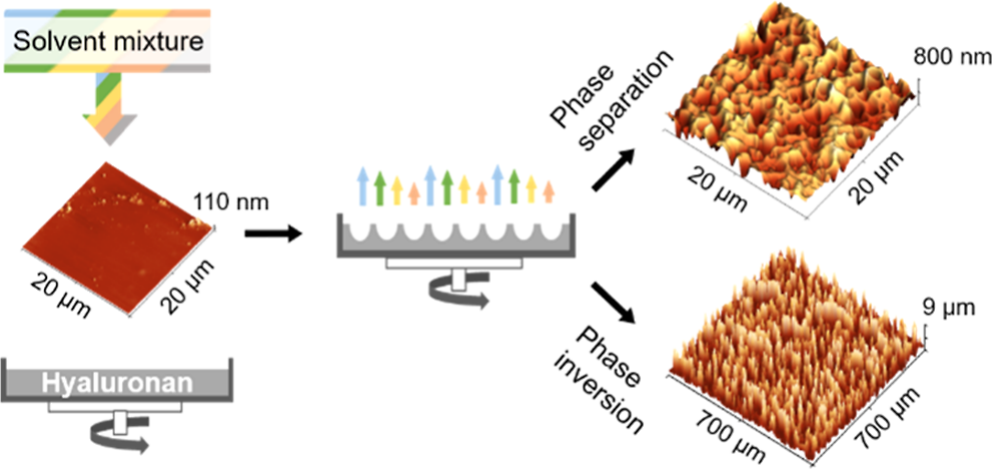

This work explores application of phase separation phenomena for structuring of films made from hyaluronan. A time-sequenced dispensing of different solution mixtures was applied under rotation of hyaluronan-covered substrates to generate surface textures. This method is applicable in direct surface modification or cover layer deposition. Changes in the surface topography were characterized by atomic force microscopy, optical microscopy, and contact and non-contact profilometry. The mechanical properties of the surface-modified self-supporting films were compared using a universal testing machine. Experimental results show that diverse hyaluronan-based surface reliefs and self-supporting films with improved mechanical properties can be prepared using a newly designed multi-step phase separation process without the need for sacrificial removable templates or additives.

## 1 Introduction

Surface modifications are among the most important material post-processing techniques utilized in various industries. These modifications can be achieved either by a changing original material surface topography or a deposition of cover layers.

Various methods can be used to prepare defined surface textures, including template molding,^1−3^ colloidal particles,^4^ polymer mixtures,^5,6^ and photopolymerized layers.^7,8^ However, these methods have some disadvantages, such as the need to remove the template and the risk of surface contamination. To overcome these problems, a molding agent converting into a gas phase, after the required texture has been generated, can be used advantageously. Commonly used methods include gaseous foaming agents,^9,10^ freezing approaches,^11^ and solvent mixtures inducing phase separation processes.^12,13^

Phase separation methods on polymer surfaces can lead to the formation of various topographies,^13−15^ which is observed in both dry^16^ and wet casting processes.^17^ Physicochemical factors that can cause phase separation include shear forces,^18,19^ temperature changes,^20^ chemical reactions,^21^ and poor solvents.^22−24^ In this work, we employed dropping of poor and good solvent mixtures templating the resulting texture. The droplet deposition could be achieved either by condensation of poor solvent vapors on a pre-swollen polymer surface^13,25^ or sequential dropping onto a solid polymer surface.^12,26^

Amorphous synthetic polymeric systems without strong physical or chemical crosslinks^5,13,23,27,28^ can be easily textured using a mixture of solvents, as described exemplarily in our previous work.^12^ However, different situations arise in the case of polysaccharide-based materials, which reveal stabilizing the spatial arrangement of macromolecular chains as well as strong network crosslinking by hydrogen bonds. Hence, the surface modifications of biopolymers by phase separation are rather seldom in the literature. Only indirect procedures based on imprinting the desired reliefs have been described.^1,29−32^

This work aims at development of a direct phase separation texturing procedure for hyaluronan (HU)-based surfaces and self-supporting films.

This biopolymer performs a variety of functions in living organisms, e.g., in the extracellular matrix as a support for cells or in joints as a hydrating agent and lubricant.^33−36^ HU is known for its biocompatibility and finds broad applicability in biomedical, tissue engineering, and pharmaceutical research as well as in arthritis treatment, cosmetics, dermatology, plastics, and eye surgery.^37^

## 2 Experimental Section

### 2.1 Materials and Reagents

Bacterial sodium salt of hyaluronic acid of various molecular weights was purchased from Contipro a.s. (Dolní Dobrouč, Czech Republic). The average molecular weight (MW) of HU was in the range from 330 to 1000 kDa, as characterized by the AF4-MALS chromatography in our previous work.^38^ Polystyrene (PS) Petri dishes with a diameter 34 mm, sterilized by UV radiation, free from pyrogens and DNA/RNA for cell cultivation (TPP Techno Plastic Products AG, Trasadingen, Switzerland), were used as a substrate for hyaluronan layers. Ultrapure water (H_2_O, 18.2 MΩ cm), *n*-butanol (*n*BT, Sigma-Aldrich Ltd., St. Louis, MO, USA), isopropyl alcohol (IPA, Sigma-Aldrich Ltd., St. Louis, MO, USA), tetrahydrofuran (THF, HPLC grade; Sigma-Aldrich Ltd.), 2-ethoxyethanol (ETH, p.a.; Sigma-Aldrich Ltd.), and dimethylsulfoxide (DMSO, Sigma-Aldrich Ltd., St. Louis, MO, USA) were used as received.

### 2.2 HU Solution Preparation

The dissolution of HU was carried out at 50 °C on a magnetic stirrer for 24 h. Aqueous solutions with a HU content of 3 wt % for a mean MW = 330 kDa, 3 wt % for a mean MW = 470 kDa, and 0.7 wt % for a mean MW = 1000 kDa were prepared.

### 2.3 Preparation and Characterization of Structured Substrates

#### 2.3.1 HU Coating Preparation

Before the HU coating, the surface of the PS dishes was modified to increase the adhesion of the solubilized HU films using previously described method.^12^ Briefly, the polystyrene Petri dish surface was treated with 5 doses of 200 microliters of the THF/ETH mixture in a ratio of 1.5:8.5. The time delay between individual doses was 5 s. After the last dose, the sample was left to rotate for additional 120 s. All samples were modified at 298 K (substrate, solutions, and surrounding atmosphere) and air humidity of 50%.

The microporous PS surface was further hydrophilized by air plasma in a Diener Femto plasma reactor with a capacitive coupling at the frequency of 13.56 MHz (Diener electronic GmbH + Co.KG), the air pressure of 1 mbar, and the air flow rate of 5 sccm (purity 99.999%). The forward power was set to 100 W, and the reflected power was maintained at 10% with the help of the matching circuit during all experiments. The plasma processing time was 60 s. After treatment, the substrates were kept at a constant temperature of 298 K in a desiccator. Control experiments showed that the surface microstructure in comparison to the plain PS substrate did not affect the type of texture formed on the HU film later, as the thickness of the solidified HU layer was 30 times higher than the depth of micropores on the PS surface.

For coating of PS dish surface preparation, 5 g of HU water solution was cast onto a plasma-activated, hydrophilized PS dish (*d* = 34 mm). Solidification of the HU solution in the PS dish was carried out at 50 °C in the laboratory dryer (Memmert GmbH, Schwabach, Germany) without forced air circulation for 24 h. Before the texturing with solution mixtures, the samples were stored for 24 h in a desiccator at a laboratory temperature of 23 °C.

#### 2.3.2 Texturization of HU Coatings

##### 2.3.2.1 Texturization by Phase Separation

For the preparation of the textured HU surfaces by phase separation ultrapure water, the various ratios of the components in the modification mixture (*n*BT/IPA; 3.25:6.75, 3:7, 2.75:7.25), various numbers of 200 μL doses (0–40×), dosing sequence (2.5–10 s), and sample rotation speed of 1600 or 2100 rpm were used. After deposition of the last dose of the modifying mixture, the sample was rotated for another 120 s at a temperature of 23 °C and relative humidity 50%. After drying the sample surface, and evaporating the used solvents, the surfaces were stored at 23 °C in a desiccator.

##### 2.3.2.2 Texturization by Phase Inversion

For the preparation of the textured HU surfaces by phase inversion two different modification mixtures [type A: 1.5 *n*BT: 3.5: IPA: 1.1H_2_O: 0.5 DMSO: 2.3 HU solution (3 wt % in H_2_O, MW = 470 kDa); type B: 1.5 mL *n*BT: 3.5 mL IPA: 1.1 mL H_2_O: 0.5 mL DMSO: 1.6 mL HU solutions (3 wt % in H_2_O, MW = 470 kDa) and 0.6 mL HU solutions (1.5 wt % in H_2_O, MW = 1000 kDa)] were used. Ten doses, each 300 μL of the mixed solution type A or type B, were dispensed onto the surface of HU (MW = 470 kDa) with a time interval of 40 s at a temperature of 23 °C, relative humidity 50%, and a sample rotation speed of 2100 rpm. After deposition of the last dose of the modifying mixture, the sample was rotated for another 120 s and then dried at 50 °C for 15 min. After drying the sample surface and evaporating the used water in the modification mixture, the surfaces were stored at 23 °C in a desiccator for 24 h.

### 2.4 Sample Characterization and Analysis

#### 2.4.1 Atomic Force Microscopy

The surface topography of structured surfaces was characterized with an atomic force microscope, model NTEGRA-Prima (NT-MDT, Moscow, Russia). Measurements were performed at the scan rate 1 Hz, with a resolution of 512 × 512 pixels in tapping mode at 298 K under the air atmosphere. A silicon-nitride probe NSG01 (AppNano, Mountain View, CA, USA) with a resonant frequency of 150 kHz and a stiffness constant of 5.5 N m^–1^ was used. The data from AFM were processed in the Gwyddion 2.55 software (D. Nečas, P. Klapetek, Czech Metrology Institute, Jihlava, Czech Republic).

#### 2.4.2 Contact Profilometry

The changes in the surface topography and roughness for all samples were characterized by the contact profilometer, model DektaXT (Bruker, Billerica, MA, USA). A tip with a radius of curvature of 2.5 μm and a pressure equivalent of 3 mg was used. The surface roughness values (Ra) and maximum height changes (Rz) were determined from five individual measurements according to the SME B46.1 standard.

#### 2.4.3 Optical Profilometry

A 3D optical microscope Contour GT-K (Bruker, Billerica, MA, USA) based on white light interferometry with use of 20× objective lenses were used to visualize surface topography. The resulting topography maps were processed in the Gwyddion 2.55 software.

#### 2.4.4 Optical Microscopy

The dried polymer layers were characterized by optical microscope Nikon Eclipse 50i (Nikon, Minato, Japan).

#### 2.4.5 Mechanical Testing

The Instron 3345J8169 (Instron, Norwood, Massachusetts, USA) universal testing machine was used to determine the tensile strength of the prepared HU layers. The HU films were cut to the required shape according to the ISO standard [ISO 20753:2018(E)]. Prior to tensile testing of the selected HU samples, the films were stored in a desiccator with silica gel at 23 °C for a minimum of 7 days so that the ratio of residual water in the measured materials was comparable.

A force transducer with a maximum measuring range of up to 100 N and accuracy 10^–3^ N was used. The crosshead speed was 10 mm/min. The measurements were carried at 23 °C and 50% relative humidity.

#### 2.4.6 Image Processing and Analysis

Images were processed and analyzed using the ImageJ software, version 1.5 (W. Rasband, National Institutes of Health, Bethesda, MD, USA), and the scale bars were added.

The presented results are based on the analysis of at least 10 individual samples in each experiment variation. A detailed image analysis procedure is included in Supporting Information.

## 3 Results and Discussion

### 3.1 Texturing of the HU Surface by Phase Separation

**Figure 1:**
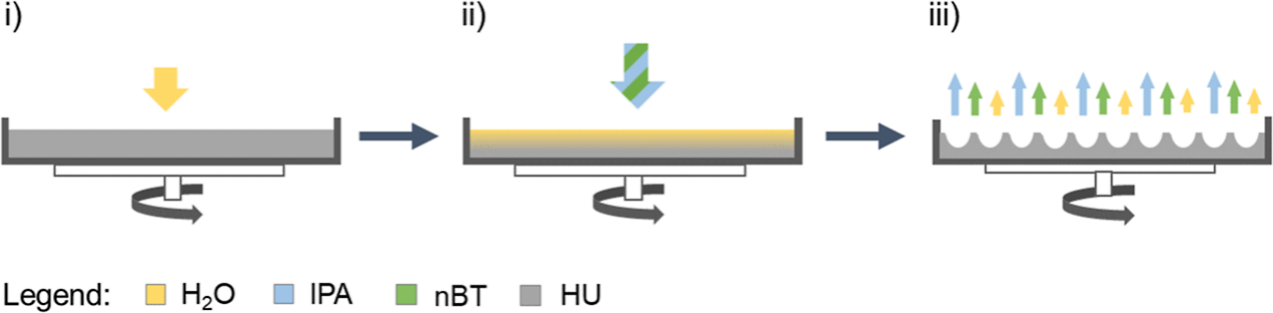
Principle of HU surface texturization using phase separation associated with different evaporation rates of good and poor solvents during the rotation. (i) Swelling of the surface with dosing of good solvent H_2_O; (ii) deposition of texturing mixture containing poor solvent mixture of IPA and *n*BT; and (iii) surface texture solidification after solvent evaporation.

The preparation of textures on the HU cast films was initially inspired by the principles we described for the PS surface recently.^12^ In contrast to the phase separation approaches described in the literature,^13,23,26,39^ this procedure is unique especially by the gradual, time-sequenced two-step dosing of a one-component good solvent and multicomponent poor solvent mixture onto a rotating HU film, as illustrated in Figure 1.

In this work, HU surfaces were treated in the first step with good solvent water (Figure 1i), followed by the mixtures of IPA/*n*BT at different ratios (Figure 1ii). The overall texturing process can be described as follows: initial swelling and partial dissolution of the HU surface with water; IPA/*n*BT diffusion into HU with H_2_O; growth of the surface-swollen layer; phase separation of *n*BT due to fast evaporation of IPA; embossing of *n*BT microdroplets into the softened HU surface; reforming (aggregation) of *n*BT microdroplets; gradual evaporation of remaining H_2_O and *n*BT; and surface relief solidification. Although water swells and partially dissolves the HU surface, to keep the description simple in the following discussion, only the term swelling will be used.

Water was chosen as a good solvent for HU due to its non-toxic nature and natural role in the polysaccharide hydration. IPA was chosen as a HU precipitant, commonly used in manufacturing practice and due to its unrestricted miscibility in aqueous media. However, due to its rapid evaporation relative to water, this poor solvent cannot be used as a pore-forming component. For these purposes, *n*BT was chosen due to its miscibility with IPA and slower evaporation rate relative to water containing HU. Apart from IPA and *n*BT, a number of other solvents have been unsuccessfully tested (2-ethoxyethanol, ethanol, and methanol); hence, they were not considered in this work.

Based on previous research^12,40−42^ as well as additional studies,^13,23^ we hypothesized that the characteristics of the resulting surface texture could be influenced by various factors such as temperature, rotational speed, and IPA/*n*BT ratio. These factors will be further discussed.

**Figure 2:**
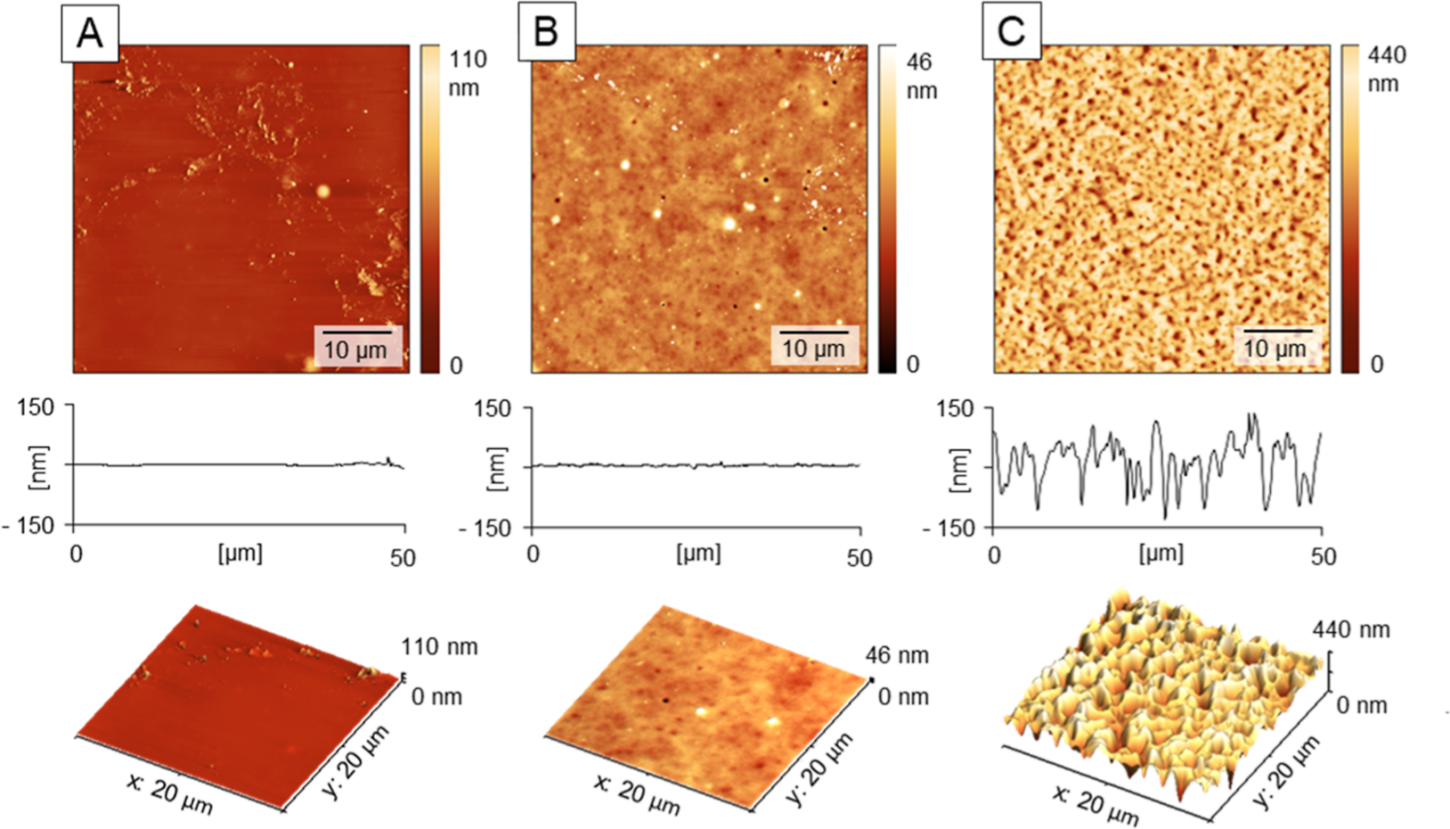
Effect of the HU film swelling on micro-texturing. The film forming HU with MW = 330 kDa was compared after surface pretreatment with water: (A) 0 μL, (B) 2×, and (C) 4 × 200 μL and surface structuring with 25 × 200 μL of a 3:7 mixture of *n*BT and IPA, dosing sequence 5 s. All modification steps were carried out at rotation speed 2100 rpm. Data from AFM heights signals.

For successful surface texturing, it was essential to understand the effect of water in the HU layer. Figure 2 shows AFM images comparing modified surfaces with and without prior surface swelling. The experimental results showed that the HU surface cannot be effectively modified without initial swelling with water, as shown in Table 1. In addition, it is necessary to limit this swelling to a certain extent so that the polymer is not washed off the modified surface. Therefore, a multi-step procedure coupled with time-sequenced dosing of water and mixed solvents was chosen. For the initial swelling of the HU surface, a minimum of 2 × 200 μL H_2_O should be deposited on the rotating surface at 5 s intervals. As the number of H_2_O depositions increases, the thickness of the surface layer increases and the depth of the pores formed in the second step increases. After exceeding four consecutive doses of swelling agent (H_2_O), there is no further increase in the depth of the pores formed. This is due to the gradual washing away of the top, mobile HU layer during the subsequent modification steps consisting of dosing a mixture of *n*BT and IPA in a 3:7 volume ratio. More detailed results of the image data analysis are presented in the Supporting Information.

**Table 1:** Image Analysis of the Surface Profiles in Figure 2^a^

pores analysis	A	B	C
number of pores	0	15 ± 2	356 ± 12
pores covered area [μm^2^]		1.8 ± 0.3	55 ± 2
average pore area [μm^2^]		0.14 ± 0.02	0.15 ± 0.01
average pore diameter [μm]		0.34 ± 0.04	0.34 ± 0.01

a Detailed roughness and porosity analysis see in Table S1.

**Figure 3:**
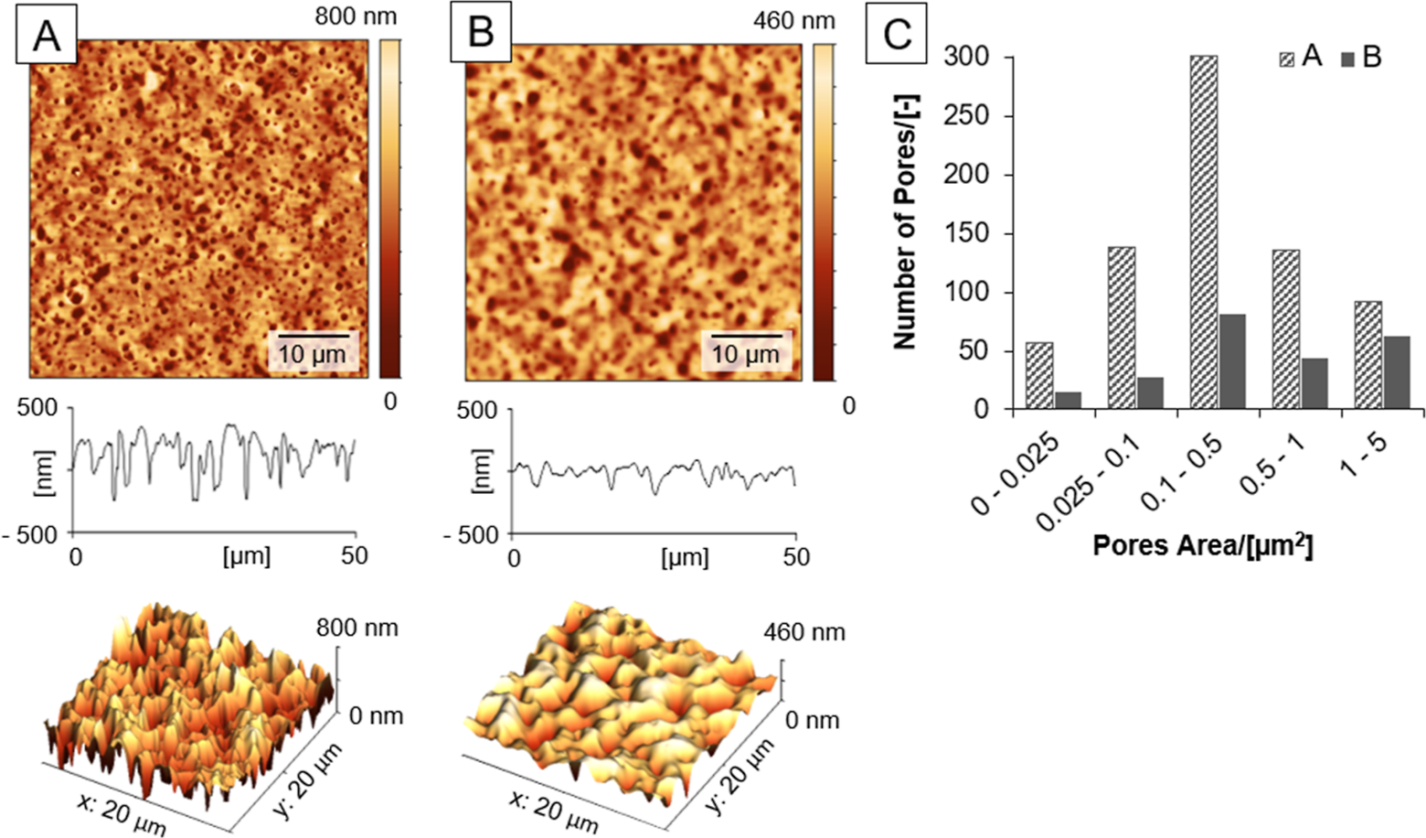
Effect of water content in the HU layer (MW = 470 kDa) on the formation of porous textures in case of (A) pre-**dried HU film** and (B) pre-**swollen HU film**. Surface modification sequence was the same: 4 × 200 μL water doses and 40 × 200 μL doses of a 3:7 *n*BT/IPA mixture, dosing sequence 5 s. The surfaces were visualized using AFM (2D and 3D representations) and profile sections and analyzed for distribution of pores after the treatments in (C). All modification steps carried out at rotation speed 2100 rpm. Data from AFM.

The rate of the HU surface swelling by water depends on the residual amount of water in the HU layer. Water acts as a good solvent for this type of polymer and also as a strong plasticizing agent. For this reason, two types of samples with different residual amounts of water were further modified. The sample with a high moisture content was pre-swollen at elevated humidity (100%) for 24 h. The weight of this sample increased by more than 130% compared to the pre-dried sample, which was kept in a desiccator with silica gel for at least 7 days. From the results shown in Figure 3 and Table 2, more pronounced porous structures appeared clearly in the case of the films with the lower water content, as shown in Figure 3A. The high residual water content of the polymer matrix, together with a multiple fold swelling (4 × 200 μL H_2_O, dosed in 5 s intervals), resulted in a viscous surface layer that can be easily washed to the edge of the rotating dish, resulting in shallower pores, as shown in Figure 3B.

**Table 2:** AFM Image Analysis of the HU Surfaces in Figure 3^a^

pores analysis	pre-dried (A)	pre-swollen (B)
number of pores	752 ± 21	255 ± 29
pores covered area [μm^2^]	288 ± 15	210 ± 9
average pore area [μm^2^]	0.38 ± 0.06	0.84 ± 0.06
average pore diameter [μm]	0.51 ± 0.04	0.78 ± 0.03

a Detailed roughness and porosity analysis see in Table S2.

**Figure 4:**
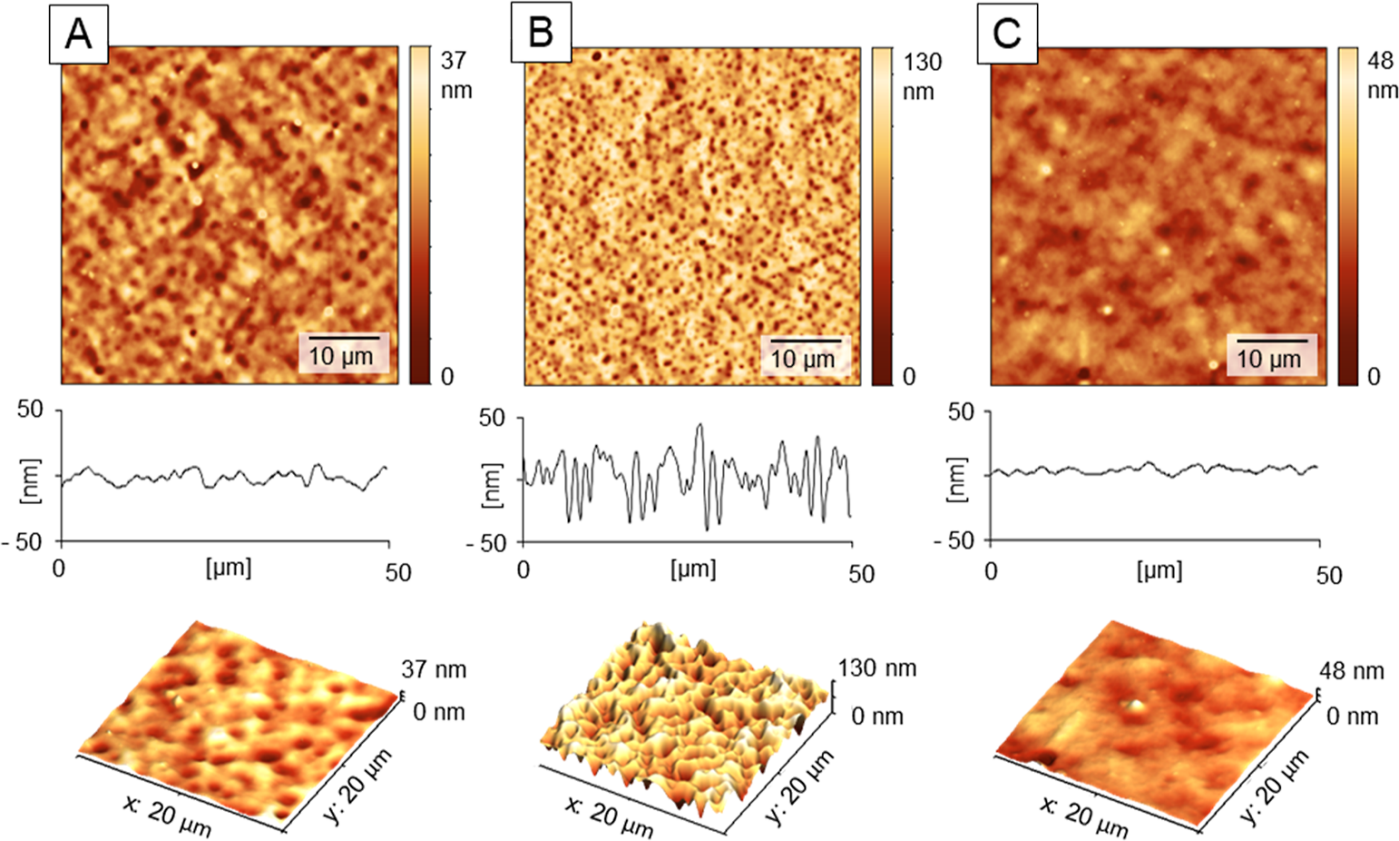
Effect of the alcohols ratio on the HU film (MW = 330 kDa) texture after surface pretreatment with 3 × 200 μL water and texturing with 10 × 200 μL nBT/IPA in ratio (A) 3.25:6.75, (B) 3:7, and (C) 2.75:7.25, dosing sequence 5 s. All modification steps carried out at rotation speed 2100 rpm. Data from AFM heights profiles.

Another important variable is the choice of the alcohol mixture. Experiments showed that the most suitable combination is the mixture of *n*BT and IPA 3:7 with a minimum water content (0.3 wt %). It was found that as little as 2.5 wt % change in the relative ratio of *n*BT to IPA inhibited the formation of pronounced porous texture, as shown in Figure 4 and Table 3.

**Table 3:** AFM Image Analysis of the HU Surfaces in Figure 4^a^

pores analysis	A	B	C
number of pores	40 ± 7	163 ± 5	2 ± 1
pores covered area [μm^2^]	14 ± 3	23 ± 3	1.2 ± 0.2
average pore area [μm^2^]	0.5 ± 0.1	0.15 ± 0.01	0.56 ± 0.05
average pore diameter [μm]	0.60 ± 0.05	0.34 ± 0.01	0.47 ± 0.02

a Detailed roughness and porosity analysis see in Table S3.

**Figure 5:**
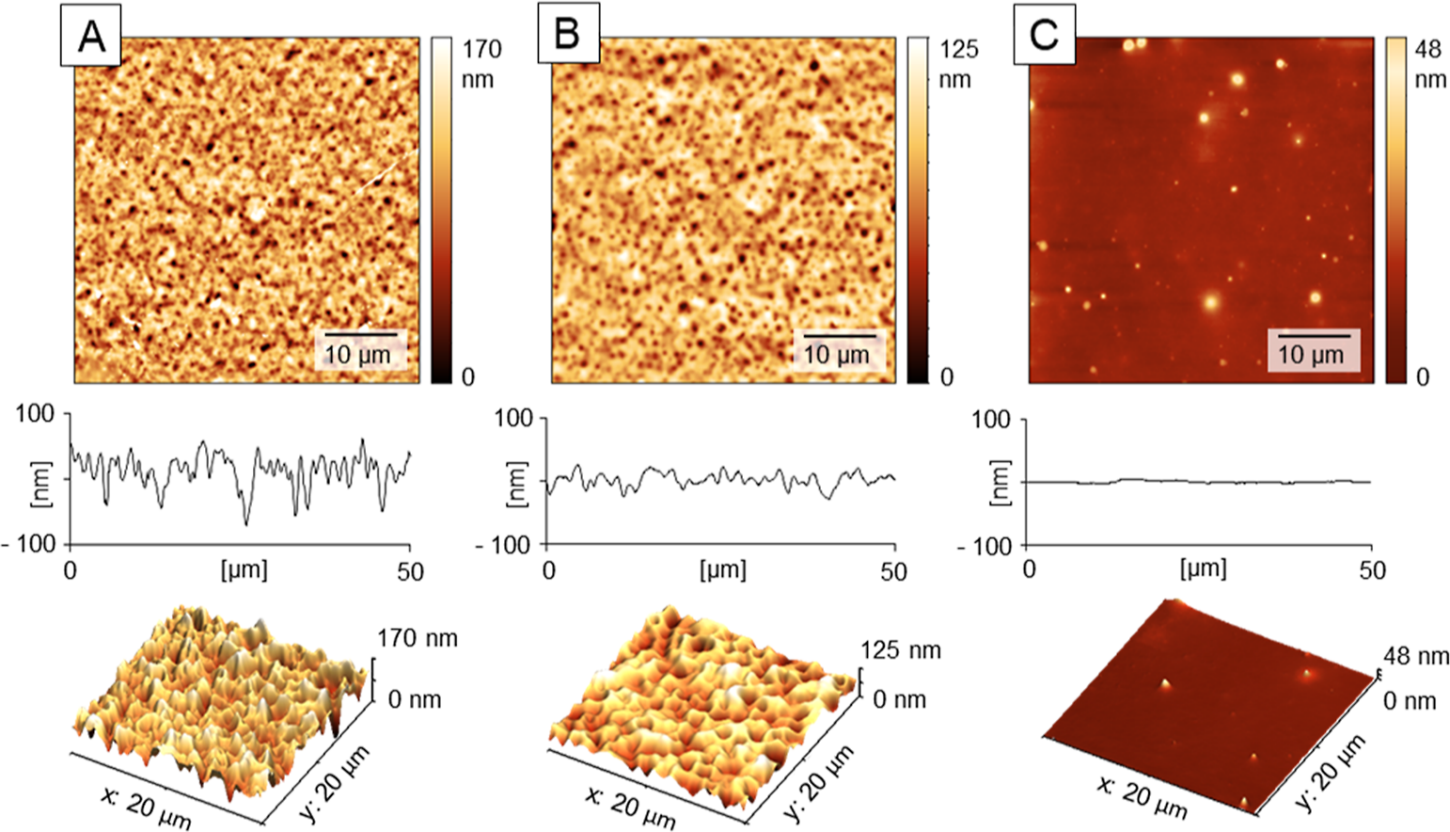
Effect of the dosage frequency on the surface microtexture. HU films (MW = 330 kDa) were textured using 3 × 200 μL of water followed by 10 × 200 μL of nBT/IPA in the ratio 3:7, at variable dosing times: (A) 2.5 s, (B) 5 s, and (C) 10 s. All modification steps were carried out at rotation speed 2100 rpm. Data from AFM heights profiles.

Experiments devoted to study the effects of the *n*BT/IPA dosing showed that the HU-based porous surface could be prepared only in the case of short intervals (2.5 or 5 s). In the case of the prolonged dosing sequences (10 s or more), solvent evaporation and repeated “curing” of the HU surface occurred, which prevented the distinct porous textures, as shown in Figure 5 and Table 4.

**Table 4:** AFM Image Analysis of the HU Surfaces in Figure 5^a^

pores analysis	A	B	C
number of pores	173 ± 23	123 ± 5	0
pores covered area [μm^2^]	30 ± 3	27 ± 5	
average pore area [μm^2^]	0.17 ± 0.01	0.22 ± 0.03	
average pore diameter [μm]	0.36 ± 0.01	0.41 ± 0.04	

a Detailed roughness and porosity analysis see in Table S4.

Similar to the PS surfaces,^12^ the type of HU surface texture depended also on the samples’ rotation speed. A rotational speed of 2100 rpm was used to modify the HU-based surfaces presented here. Lower rotation speed of 1600 rpm resulted in the solvent accumulation, causing more intense swelling, dissolution, and removal of material for the HU surface, resulting in profiles similar to Figure 3B.

**Figure 6:**
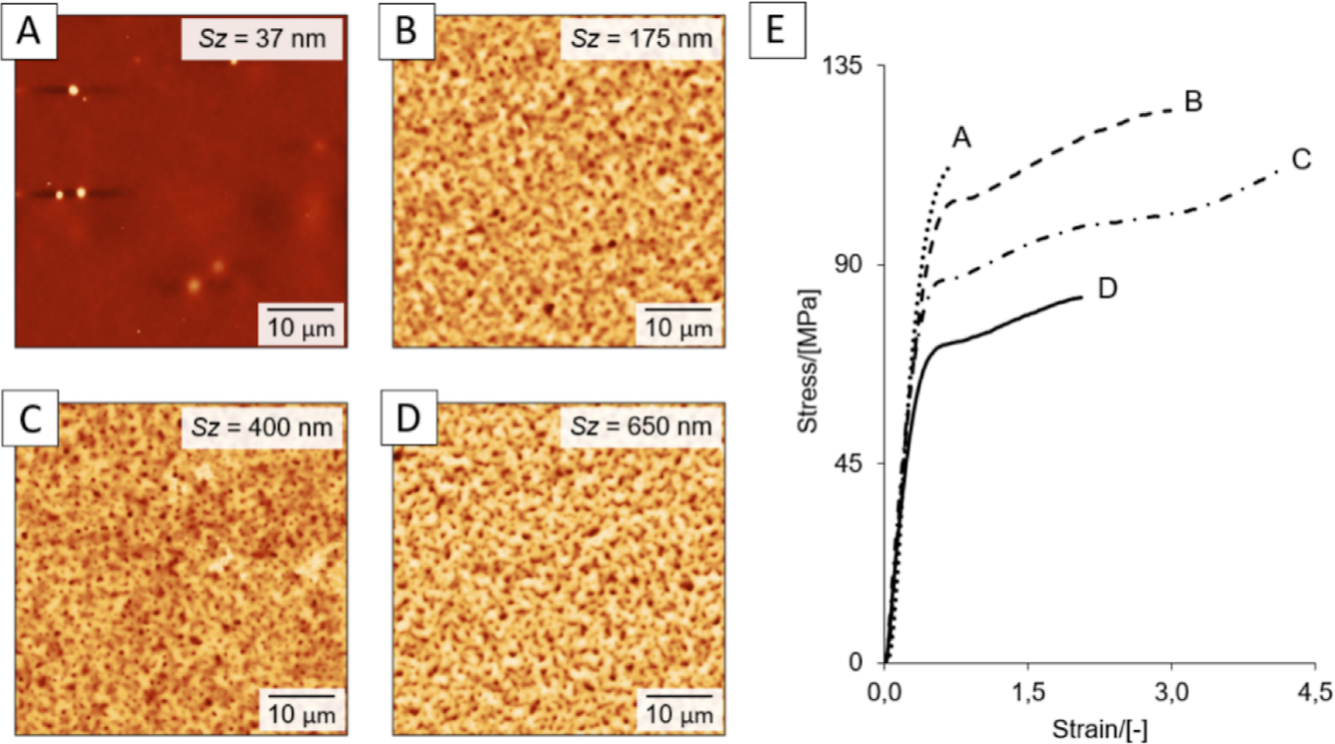
AFM images of HU films (MW = 330 kDa) without (A) and with (B–D) surface treatments before tensile testing: using doses of 200 μL water and 200 μL of *n*BT/IPA 3:7 mixtures. The doses were (B) 3× water and 20× alcohols mixture, (C) 4× water and 30× alcohols mixture, and (C) 5× water and 40× alcohols mixture, dosing sequence 5 s. All modification steps carried out at rotation speed 2100 rpm. Images from AFM. (E) Tensile curves of self-supporting films from (A–D). *Sz* indicates the sum of the largest peak height and the largest pit depth values within the defined area.

Interestingly, tensile tests of self-supporting HU-films showed that the formation of surface pores was associated with up to a fourfold increase in elongation. This increase is conditioned by the action of the modifying mixture based on *n*BT with IPA and by the number of modification steps, as shown in Figure 6.

The techniques used to prepare textured HU surfaces via the phase separation method were highly consistent, resulting in porosity covering 80% of the area. The analysis excluded the peripheral regions near the Petri dish edges due to solvent accumulation causing artifacts.^12^

### 3.2 Phase Inversion Modification of the HU Surface

Similar to the case of PS,^40^ the addition of HU to the modification solution allowed for preparation of new surface texture types. From a physicochemical point of view, this approach corresponds to the phase inversion described in the literature.^43−45^ Due to the structural and conformational differences between PS and HU, the modification process of the polysaccharide was much more complex and did not allow for the preparation of bulk porous structures as in the case of synthetic polymers.^40^ A key point in the modification was to find out a suitable ratio of the components of the mixture, correct surface rotation speed, amount and volume of the dosed mixture, dose frequency, and especially content of residual water in the HU pre-swollen layer.

**Figure 7:**
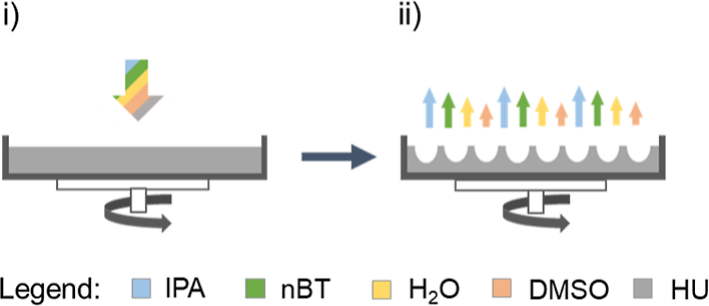
Principle of HU surface texturization using phase inversion under conditions of different evaporation rates of good and poor solvents during the substrate rotation. (i) Deposition of modification mixture containing IPA + *n*BT + H_2_O + DMSO + HU; (ii) surface texture solidification after the solvent evaporation.

Using the phase separation method of the *n*BT/IPA mixture, it was possible to prepare surface reliefs with *S*_max_ in the submicrometer range, as can be seen in the previous chapter (Figures 2, 3, 4, and 5). In the case of the phase inversion using the mixture with dissolved HU, profiles with heights in micro-to submillimeter range were observed. To prepare these textures, it was necessary to mix water, HU solution, and eventually dimethyl sulfoxide (DMSO) to the *n*BT/IPA-based modification solution, as shown in Figure 7.

**Figure 8:**
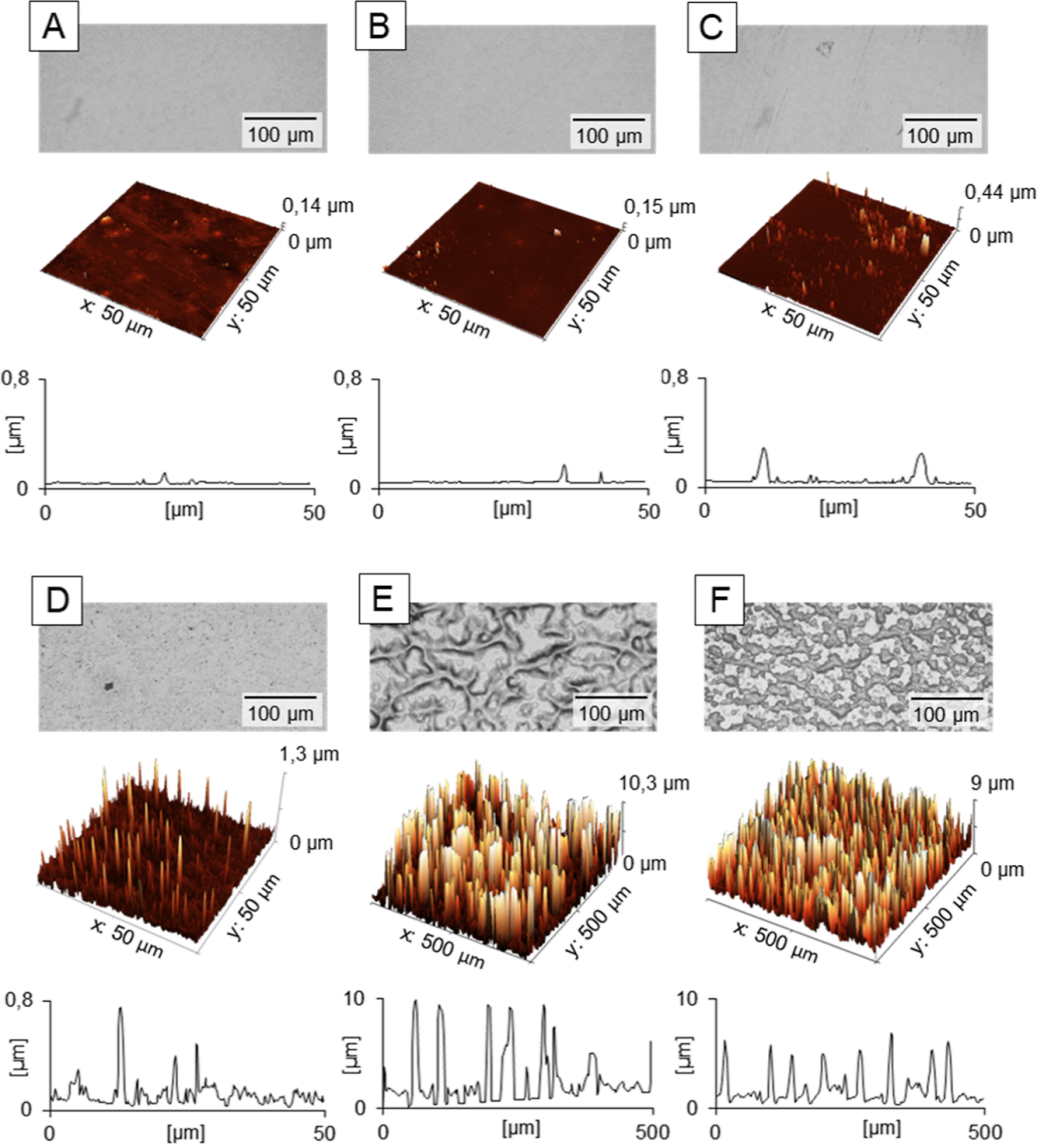
Effect of the modification mixture (type A) composition on the generation of microstructured surfaces topography. The HU film (MW = 470 kDa) were modified with (A) *n*BT/IPA (3:7); (B) *n*BT/IPA/H_2_O (3:7:1.1); (C) *n*BT/IPA/H_2_0/DMSO (3:7:1.1:0.5); (D) *n*BT/IPA/**HU** (3:7:2.3); (E) *n*BT/IPA/H_2_O/**HU** (3:7:1.1:2.3); (F) *n*BT/IPA/H_2_0/DMSO/**HU** (3:7:1.1:0.5:2.3), where **HU** represents 3 wt % HU (MW = 470 kDa) solution in water. The scans were acquired with optical microscope (upper panel), AFM heights sensor (middle panel), and optical profilometer (lower panel).

The addition of DMSO to the solution results in different intra- and interchain interactions between polysaccharide’s macromolecular chains, as Scott described^36,43^ in comparison to water. Experimental observations showed that the added H_2_O acts as a swelling agent, while DMSO increases the aggregation rate of HU with IPA around the “pore-forming” component (*n*BT), as shown in Figure 8.

**Figure 9:**
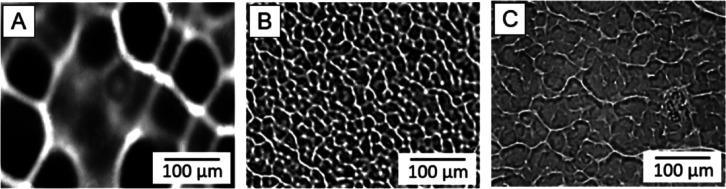
Effect of HU molecular weight in the modification mixture and for the texturing of HU films. Following MWs of HU in the film and in the modification mixtures were used: (A) 1000 kDa (film): 470 kDa (mixture type A); (B) 470 kDa (film): 470 kDa (mixture type A); (C) 470 kDa (film): 470 kDa plus 1000 kDa (mixture type B). The micrographs were acquired with an optical microscope.

The drying of a hydrophilic water swollen HU film is an order of magnitude slower compared to the PS surface, which dissolved in rapidly evaporating agents such as tetrahydrofuran.^12^ Hence, it was crucial to take into consideration mean MWs of the HU in the modification mixture and in the treated surface. The average MWs of HU were characterized by the AF4-MALS chromatography, as described in detail in the Supporting Information of our previous work.^38^ With widely different MWs, the aggregation capacity of the system, the swelling rate, and the thickness of the swollen layer caused the coalescence of the pore-forming component (*n*BT), as shown in Figure 9A,C. Moreover, the surface swelling rate is influenced by the residual amount of water in the HU film before the treatment, which implied tight control of the film drying before the coating procedure, as described in HU Coating Preparation.

Unlike in the phase separation technique, in the case of HU phase inversion, it was necessary to use a long deposition time (20 to 60 s) between the doses of the modification solution. This is due to the ability of HU to bind large amounts of water and its slow evaporation compared to alcohol-based organic solvents.

## 4 Conclusions

A simple, fast, clean, and highly reproducible phase separation procedure for the preparation of textured surfaces based on hyaluronan using a mixture of good and poor solvents was developed.

It has been found that the hyaluronan films have to be pre-swollen prior the modification. Subsequently, a modification mixture with a narrow ratio of *n*BT and IPA should be deposited. Moreover, the outcome of the modification process depends on the residual water in the modified polymer film determined by its preparation and storage. We found also narrow windows for the dosage frequency of the modification solution as well as for the rotation speed of the substrate in which the desired surface changes can be induced. The modification solution must be deposited on the HU surface in short intervals (2.5 to 5 s) with many repetitions (10 to 40 doses) and rotation close to 2100 rpm. The process is ideally suited for the production of microporous surfaces with relief heights in the submicrometer range.

For preparing surface irregularities in order of micrometer up to submillimeter range, a phase inversion approach can be used. Here, incorporation of the aqueous hyaluronan solution to the modification mixture based on organic good and poor solvents as well as the prolonged dosing interval is required for the texture solidification. Furthermore, the molecular weight distribution of the hyaluronan used in both the film layer and in the modification solution has a major influence on the formation of the specific surface structure.

The modification method helped to improve its mechanical properties. Materials with a textured surface were more ductile compared with a rather brittle smooth hyaluronan film. Hence, the developed texturing could be employed for the preparation of drug carriers with improved mechanical properties and accelerated solubility.

## Supporting Information Available

Detailed analysis of surface roughness of Figures 2–5 (PDF)
