# Supplementary material for: Controlled Structuring of Hyaluronan Films by Phase Separation and Inversion
Source: Langmuir. 2023 Sep 1;39(37):13140–8. doi: 10.1021/acs.langmuir.3c01547 (PMC10515624; doi:10.1021/acs.langmuir.3c01547)
Supplement: Supplementary file 1 — la3c01547_si_001.pdf [file la3c01547_si_001.pdf]

Supporting Information

Controlled structuring of hyaluronan films by phase separation and inversion

*Petr Smolka, Markéta Kadlečková, Karolína Kocourková, Martina Bartoňová, Filip Mikulka, Eliška Bognerová, Aleš Mráček, Lenka Musilová, Martin Humeník, Antonín Minařík**

† Department of Physics and Materials Engineering, Tomas Bata University in Zlín, Vavrečkova 5669, 760 01 Zlín, Czech Republic

‡ Centre of Polymer Systems, Tomas Bata University in Zlín, Třída Tomáše Bati 5678, 760 01 Zlín, Czech Republic

§ Department of Biomaterials, Faculty of Engineering Science, Universität Bayreuth, Prof.-Rüdiger-Bormann.Str. 1, 95447 Bayreuth, Germany

* Corresponding author: E-mail: minarik@utb.cz, +420 576 03 5086, ORCID: 0000-0002-0055-675X

Image analysis of surfaces

Data obtained by atomic force microscopy (AFM) (NTEGRA Prima, NT-MDT) were processed using the the Gwyddion – Free SPM data analysis software, version 2.60 (D. Nečas, P. Klapetek, Czech Metrology Institute, Czech Republic)
The roughness and porosity analysis was performed by tools in Gwyddion software using the original AFM image size of 50×50 μm.

Roughness

Roughness parameters were obtained by Profile Extraction Tool. Cross-sections were extracted for each original image and the roughness parameters were calculated:
Ra [nm] – arithmetical mean height of a line,
Rq [nm] – root mean square average of the profile heights over the evaluation length,
Skeweness [-] – measure of the average of the first derivative of the surface,
Kurtosis [-] – measure of whether the data are heavy-tailed or light-tailed relative to a normal distribution.

Area Roughness

Area Roughness parameters were calculated from original image by Statistical Quantities Tool in Gwyddion software.
Sa [nm] – area average roughness,
Sq [nm] – root mean square value of ordinate values within the definition area.

Porosity Analysis

For each original image, a slice plane was selected and interleaved with a mask that most of the pores were captured (Figure S1). The subsequent porosity analysis was then obtained by Grain Statistics Tool in Gwyddion software.

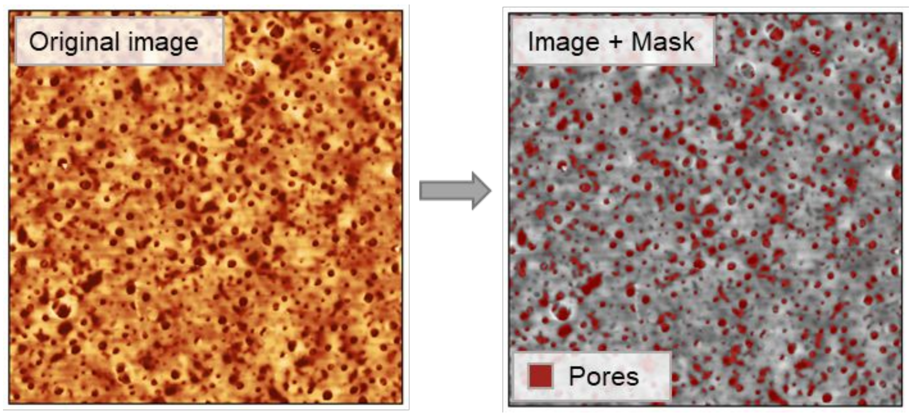

Figure S1. Left – original image from AFM (50×50 μm). Right – image with highlighted pores from which the porosity analysis was done.

Table S1. Roughness and porosity analysis for the cases in Figure 2.

	Pores Analysis	A	B	C
Roughness	Ra [nm]	2.2 ± 0.3	1.8 ± 0.1	44 ± 1
	Rq [nm]	4.3 ± 0.2	2.5 ± 0.3	57 ± 2
	Skewness [-]	2.5 ± 0.2	1.4 ± 0.5	-0.8 ± 0.2
	Kurtosis [-]	25 ± 12	12 ± 6	1.2 ± 0.6
Area Roughness	Sa [nm]	2.7 ± 0.3	2.4 ± 0.4	29.7 ± 0.8
	Sq [nm]	6.0 ± 0.4	3.4 ± 0.1	38 ± 1
	Skewness [-]	4.7 ± 0.5	0.9 ± 0.3	-1.28 ± 0.03
	Kurtosis [-]	40 ± 8	13 ± 1	1.0 ± 0.2
Pores Analysis	Number of Pores [-]	0	15 ± 2	356 ± 12
	Pores Covered Area [μm^2]	-	1.8 ± 0.3	55 ± 2
	Average Pore Area [μm^2]	-	0.14 ± 0.02	0.15 ± 0.01
	Average Pore Diameter [μm]	-	0.34 ± 0.04	0.34 ± 0.01

Table S2. Roughness and porosity analysis for the cases in Figure 3.

	Pores Analysis	A	B
Roughness	Ra [nm]	103 ± 7	49.0 ± 0.7
	Rq [nm]	131 ± 5	69 ± 4
	Skewness [-]	-0.7 ± 0.2	-0.8 ± 0.1
	Kurtosis [-]	0.7 ± 0.4	1.5 ± 0.3
Area Roughness	Sa [nm]	56 ± 1	36 ± 1
	Sq [nm]	65 ± 1	43.4 ± 0.7
	Skewness [-]	-0.33 ± 0.07	-0.9 ± 0.1
	Kurtosis [-]	-0.6 ± 0.4	-0.123 ± 0.1
Pores Analysis	Number of Pores [-]	752 ± 21	255 ± 29
	Pores Covered Area [μm^2]	288 ± 15	210 ± 9
	Average Pore Area [μm^2]	0.38 ± 0.06	0.84 ± 0.06
	Average Pore Diameter [μm]	0.51 ± 0.04	0.78 ± 0.03

Table S3. Roughness and porosity analysis for a set of samples in Figure 4.

	Pores Analysis	A	B	C
Roughness	Ra [nm]	3.8 ± 0.4	11 ± 1	2.5 ± 0.2
	Rq [nm]	5.1 ± 0.5	15 ± 2	3.1 ± 0.3
	Skewness [-]	-0.7 ± 0.2	-0.57 ± 0.04	-0.35 ± 0.15
	Kurtosis [-]	-1.32 ± 0.04	0.7 ± 0.2	-0.4 ± 0.2
Area Roughness	Sa [nm]	3.7 ± 0.2	7 ± 1	2.9 ± 0.2
	Sq [nm]	4.8 ± 0.3	9 ± 1	3.6 ± 0.4
	Skewness [-]	-0.40 ± 0.05	-1.1 ± 0.2	0.11 ± 0.05
	Kurtosis [-]	0.75 ± 0.07	0.47 ± 0.1	1.36 ± 0.09
Pores Analysis	Number of Pores [-]	40 ± 7	163 ± 5	2 ± 1
	Pores Covered Area [μm^2]	14 ± 3	23 ± 3	1.2 ± 0.2
	Average Pore Area [μm^2]	0.5 ± 0.1	0.15 ± 0.01	0.56 ± 0.05
	Average Pore Diameter [μm]	0.60 ± 0.05	0.34 ± 0.01	0.47 ± 0.02

Table S4. Roughness and porosity analysis for a set of samples in Figure 5.

	Pores Analysis	A	B	C
Roughness	Ra [nm]	20 ± 2	14.0 ± 0.7	1.2 ± 0.3
	Rq [nm]	26 ± 2	17 ± 1	1.8 ± 0.5
	Skewness [-]	-0.4 ± 0.2	-0.5 ± 0.2	1.8 ± 0.9
	Kurtosis [-]	1.0 ± 0.7	0.3 ± 0.2	1.1 ± 0.1
Area Roughness	Sa [nm]	9.2 ± 0.2	5.9 ± 0.6	1.6 ± 0.2
	Sq [nm]	11.0 ± 0.1	7 ± 0.6	3.0 ± 0.2
	Skewness [-]	-0.55 ± 0.06	-0.5 ± 0.3	5.2 ± 0.4
	Kurtosis [-]	-1.0. ± 0.1	3 ± 2	44 ± 4
Pores Analysis	Number of Pores [-]	173 ± 23	123 ± 5	0
	Pores Covered Area [μm^2]	30 ± 3	27 ± 5	-
	Average Pore Area [μm^2]	0.17 ± 0.01	0.22 ± 0.03	-
	Average Pore Diameter [μm]	0.36 ± 0.01	0.41 ± 0.04	-
